# Integrating traditional practices and social network visualization to prevent substance use: study protocol for a randomized controlled trial among urban Native American emerging adults

**DOI:** 10.1186/s13722-021-00265-3

**Published:** 2021-09-26

**Authors:** Elizabeth J. D’Amico, Daniel L. Dickerson, Anthony Rodriguez, Ryan A. Brown, David P. Kennedy, Alina I. Palimaru, Carrie Johnson, Rosanna Smart, David J. Klein, Jennifer Parker, Keisha McDonald, Michael J. Woodward, Ninna Gudgell

**Affiliations:** 1 RAND Corporation, 1776 Main Street, PO Box 2136, Santa Monica, CA 90407-2138 USA; 2 UCLA Integrated Substance Abuse Programs (ISAP), Semel Institute for Neuroscience and Human Behavior, David Geffen School of Medicine, 1640 Sepulveda Blvd., Suite 200, Los Angeles, CA 90025 USA; 3 RAND Corporation, 20 Park Plaza, Suite 920, Boston, MA 02116 USA; 4 Sacred Path Indigenous Wellness Center, Los Angeles, CA 90017 USA

**Keywords:** Native Americans, Traditional practices, Social networks, Motivational interviewing, Opioid use, Alcohol and marijuana/cannabis use

## Abstract

**Background:**

Nonmedical use of prescription opioids (defined as taking opioid medications for hedonic effects or in a manner other than prescribed) and the use of heroin have emerged in recent years as major public health concerns in the United States. Of particular concern is the prevalence of opioid use among emerging adults (ages 18–25), as this is a developmental period of heightened vulnerability and critical social, neurological, and psychological development. Data from 2015 show that American Indian/Alaska Native (AI/AN) people have the highest rates of diagnosis for opioid use disorders (OUDs). One recent study found that the overdose death rate among urban-dwelling AI/AN individuals was 1.4 times higher compared to those living in rural areas. To date, there are no evidence-based prevention programs addressing opioid use among urban AI/AN emerging adults that integrate culturally-appropriate strategies with evidence-based treatment. Traditions and Connections for Urban Native Americans (TACUNA) builds on our prior work with AI/AN communities across California to develop and evaluate culturally appropriate programming to address opioid, alcohol, and cannabis use among urban AI/AN emerging adults.

**Methods/design:**

In a randomized controlled trial, 18–25 year old urban AI/AN emerging adults will receive either TACUNA (n = 185), which comprises three virtual workshops utilizing motivational interviewing, social network visualization, and integrating traditional practices and a wellness circle, or one virtual culturally sensitive opioid education workshop (n = 185). We will evaluate intervention effects on primary outcomes of frequency of opioid, alcohol, and cannabis use, as well as secondary outcomes of social network characteristics and cultural connectedness, over a 12-month period.

**Discussion:**

This project has the potential to expand the range and effectiveness of opioid, alcohol, and cannabis services for urban AI/AN emerging adults by addressing the opioid epidemic and use of other substances at both the community and individual level. In addition, it provides important culturally grounded conceptual and practical information to advance the field of substance use interventions and enhance resiliency among this population.

*Trial registration*: ClinicalTrials.gov Identifier: NCT04617938. Registered October 26, 2020 https://clinicaltrials.gov/ct2/show/record/NCT04617938.

**Supplementary Information:**

The online version contains supplementary material available at 10.1186/s13722-021-00265-3.

## Background

Opioid use has reached epidemic proportions in the American Indian and Alaska Native (AI/AN) population [1–3]. Rates of opioid use and overdose deaths among AI/AN individuals have increased fourfold in the last ten years [4]. According to the most recent Centers for Disease Control and Prevention (CDC) data, AI/AN people had the second-highest overdose rate from all opioids in 2017 (15.7 deaths/100,000 population) among all racial/ethnic groups in the United States [5]. Of note, one recent study found that the overdose death rate among urban-dwelling AI/AN individuals was 1.4 times higher compared to those living in rural areas [6]. Furthermore, opioid use among the AI/AN population is often associated with dire social and medical costs [7, 8], including an increased risk of HIV, chronic medical problems, comorbid mental health problems, and suicide [9, 10]. However, few AI/AN people receive evidence-based treatments that are appropriately culturally tailored [2].

Unique risk factors may predispose urban AI/AN emerging adults to use opioids, alcohol, or other drugs. For example, many AI/AN people report experiences of acculturative stress, which are directly and indirectly associated with historical trauma throughout U.S. history, and significantly associated with poor health outcomes [11, 12]. One U.S. law that has been postulated to contribute to various health disparities among urban AI/AN people is the Relocation Act of 1956 [13]. This Act financed the relocation of AI individuals and AI families to job training centers in designated U.S. cities. Rather than establishing economic stability, large numbers of AI people who moved to urban areas became unemployed, homeless, and disconnected from their community-based support networks. This relocation appears to have contributed to an inter-generational effect whereby successive generations of urban AI/AN people continue to experience various health-related disparities [14, 15]. Furthermore, the transition to urban environments posed numerous challenges [16–18]. Traditionally, AI/AN people lived in extended families and among community networks and typically engaged in many traditional practices. Some urban AI/AN communities are closely connected; however, many are geographically and socially fragmented, making it difficult for AI/AN individuals to establish and retain close community ties [19, 20]. As a result, over 60 years after relocation began, urban AI/AN people still often feel ostracized, socially disconnected, confused about their identity, and victimized [21, 22]. This is important to recognize as AI/AN individuals have historically relied on healthy social connections and close-knit communities that placed a high priority on traditional practices and living a life in balance. Interventions that use evidence-based practices to build healthier social networks by encouraging traditional practices could help increase social connection and cultural identity among AI/AN emerging adults residing in urban areas.

To date, there are no prevention programs addressing opioid use among urban AI/AN emerging adults that integrate culturally-appropriate strategies with evidence-based treatment [2]. Community-based approaches to address AI/AN alcohol and other drug (AOD) use are crucial [23–25]. We are part of a group of investigators funded by the National Institutes of Health (NIH) to conduct research under the Intervention Research to Improve Native American Health (IRINAH) program [26]. Our work and the work of IRINAH has highlighted the importance of using a community-based participatory research approach when developing and implementing prevention and intervention programming with AI/AN people and intervening at multiple levels to address disparities [23, 24, 27–32]. Our team was one of the first groups funded as part of the IRINAH request for applications (RFA) in 2012, and has been the only research group, to date, to conduct culturally centered prevention/intervention work with AI/AN adolescents and emerging adults in urban settings.

Given our focus on collaborating with AI/AN communities to ensure both feasibility and sustainability of our interventions, we have worked closely with our community partner, Sacred Path Indigenous Wellness Center (SPIWC), our Elder Advisory Board, and state and local AI/AN organizations to understand the best ways to develop an opioid, alcohol, and cannabis prevention intervention program for urban AI/AN emerging adults. We sought to address the many challenges that urban AI/AN emerging adults face due to substance use and a disconnection from their culture and supportive networks. Our focus group and pilot test work in year 1 of this grant provided formative information on how to best integrate evidence-based programming with culture in a way that is engaging and developmentally appropriate for urban AI/AN emerging adults [33]. We conducted 13 focus groups (November 2019 through February 2020) with AI/AN emerging adults, parents of AI/AN emerging adults, and providers who work with AI/AN emerging adults (N = 91 participants: 32 young adults, 33 providers, and 26 parents). Focus groups helped us understand how to best address opioid, alcohol, and cannabis use among this age group, integrate a social network intervention to allow emerging adults to create healthy virtual social worlds, and determine traditional practices that are developmentally relevant for 18–25 year olds. We also conducted a pilot test of the workshop content with approximately six AI/AN emerging adults in each of the three workshops to evaluate the feasibility and acceptability of the intervention.

Overall, focus group and pilot test participants all discussed the enduring effects of historical trauma, how these effects can be exacerbated by urban stressors, and the challenges of cultural fragmentation and dispersed social support networks. Participants also described the way that AI/AN culture can provide resilience and healing through connection to nature, with an emphasis on balance and interconnectedness, and the positive social and psychological effects of ceremonies and prayer. Finally, participants emphasized the importance of providing culturally relevant programming to all urban AI/AN emerging adults as part of this trial. Thus, this protocol study has an active control group that is compared to a more comprehensive treatment group. This research design is similar to previous intervention work we conducted with urban AI/AN teens in which both groups received culturally appropriate treatment [34]. We describe the two interventions for the current study below.

### The present study

**Fig. 1:**
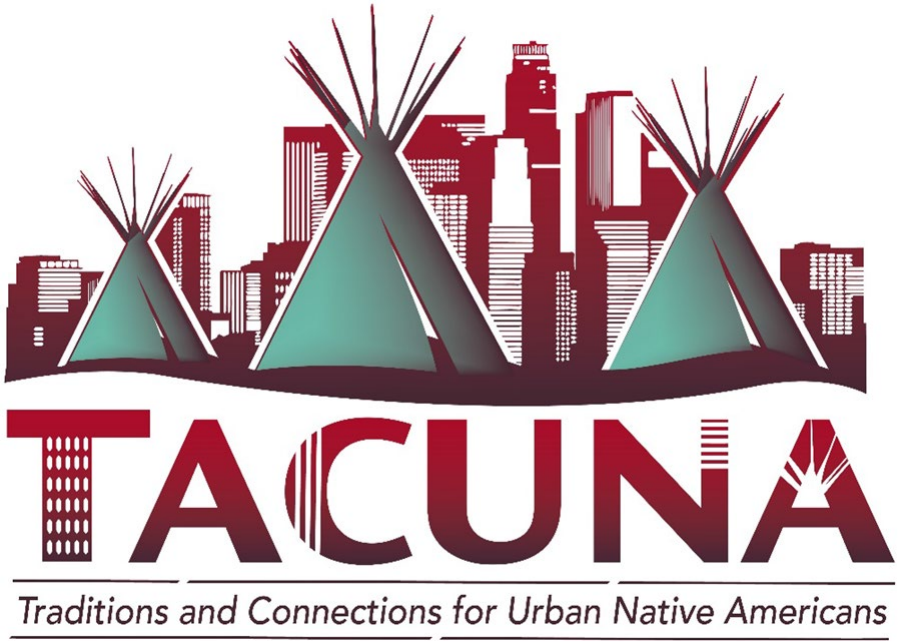
TACUNA Logo. Logo created by Robert Young (Pueblo of Acoma)

The present study was designed to address the significant gap in culturally appropriate evidence-based programming to target opioid, alcohol, and cannabis use among urban AI/AN emerging adults and to introduce social network as a crucial component of this programming. Randomized controlled trials (RCTs) are often considered the scientific “gold standard” for testing efficacy of interventions, yet, RCTs may be perceived as unacceptable and unfair in AI/AN populations because not everyone receives treatment [30]. Therefore, in this RCT, every AI/AN emerging adult will receive culturally appropriate programming as requested by the community in our focus groups [33]. Urban AI/AN emerging adults are randomized to participate in one of two cultural programs. The active control condition is an “opioid education workshop,” and the intervention program is called “Traditions and Connections for Urban Native Americans (TACUNA).” Figure 1 shows our logo, designed by Robert Young (Pueblo of Acoma). Mr. Young designed five logos for the project, which were vetted by the AI/AN community, and this logo received the most votes.

TACUNA comprises three workshops and a wellness circle. The control program comprises one culturally sensitive opioid education workshop. The primary aim is to examine the effects of these two culturally-appropriate programs on the opioid, cannabis, and alcohol use of urban AI/AN emerging adults over a one-year period. A secondary aim is to examine whether these programs increase cultural connectedness and enhance supportive social networks. Finally, we will explore potential mechanisms of change for decreases in opioid, cannabis, and alcohol use outcomes through mediation analyses, including changes in social networks and cultural connectedness.

This project also incorporates an economic evaluation, which has not commonly been done in previous intervention work with this population [35, 36]. Due to well-documented disparities in funding and availability of health services for AI/AN people relative to other Americans [37–39], the development of efficacious interventions for AI/AN emerging adults should occur alongside consideration of program efficiency and resource requirements.

In the protocol below, we focus on the primary (opioid, alcohol, and cannabis use) and secondary (social networks and cultural connectedness) aims of the project. We first describe eligibility criteria for study participants and our recruitment approach, as well as the primary and secondary outcome measures. We also discuss the format and key components of both interventions and describe the analytic plan for the primary and secondary aims, as well as the economic evaluation.

## Methods/design

### Overview

This study is part of RFA-DA-19-035, HEAL (Helping to End Addiction Long-Term) Initiative: Preventing OUD in Older Adolescents and Young Adults (ages 16–30). This study was funded as part of the National Institute on Drug Abuse (NIDA) HEAL Prevention Initiative Cooperative Agreement, comprised of nine UG3/UH3 research projects and one U24 coordinating center. This protocol focuses specifically on the TACUNA project. Due to the COVID-19 pandemic and restrictions on social gatherings, this study had to change from in-person group implementation to virtual group implementation. This allowed us to expand participation beyond our original urban sites in California as we were no longer limited by facilitating in-person groups. All study materials and procedures, including virtual implementation, have been approved by RAND’s Institutional Review Board, NIDA, and our Urban Intertribal Native American Review Board. In addition, a Certificate of Confidentiality from the National Institutes of Health protects data from subpoena. Any protocol modifications will be sent to the IRB for review, and all information will be updated as needed.

### Participants

Our interventions are targeted towards urban AI/AN emerging adults. Eligibility criteria include: (1) being between the ages of 18–25; (2) living in an urban area (i.e., not on a rancheria or a reservation); (3) self-identification as AI/AN; (4) no opioid use disorder; and (5) English speaking. We focus on 18 to 25 year olds in this study because there is a high prevalence of opioid use among all emerging adults, and this is a developmental period of heightened vulnerability and critical social, neurological, and psychological development [40, 41]. TACUNA and the culturally sensitive opioid education workshop can be used as both prevention and intervention. Thus, participants could report no substance use or some substance use. We do screen each participant; however, to determine whether they report opioid dependence, we use the Rapid Opioid Dependence Screen (RODS), an 8-item measure based on DSM-IV criteria (see “Measures”). If the participant indicates three or more affirmative answers, then they are not eligible and we provide resources and referrals to treatment programs. We will enroll 370 participants at baseline across the U.S. who are expected to represent the broader population of 18–25 year old urban AI/AN emerging adults.

### Procedures

This study uses a permuted block randomization schedule with a block size of ten. This will allow us to begin the groups once we have four emerging adults. The randomization schedule, developed in advance, ensures a balance across the opioid education workshops and TACUNA groups at all sites and at all stages of the trial, and safeguards the trial against subversion of the randomization procedure (by ensuring that the schedule cannot be guessed or changed). Given rotating admission, this also ensures we will always have enough participants in our groups throughout recruitment. We used this procedure in previous work with urban AI/AN adolescents [34], and it worked well, as teens could immediately enter a group, and groups were always full.

**Fig. 2:**
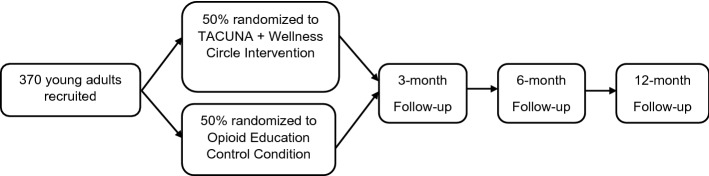
Randomized controlled trial study flow

**Fig. 3:**
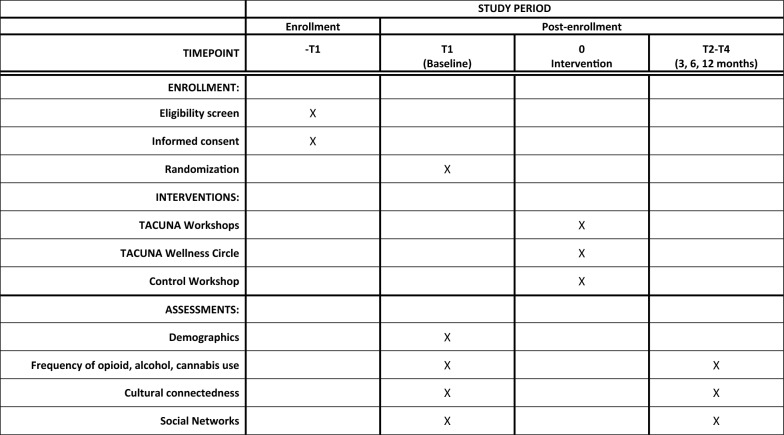
SPIRIT (Standard Protocol Items: Recommendations for Interventional Trials) flow diagram of the RCT schedule of enrollment, interventions, and assessments

Participants in the intervention condition receive TACUNA, which consists of three two-hour workshops and a wellness circle; participants in the control condition receive one culturally sensitive two-hour opioid education workshop (both described below). Recruitment occurs by advertising the study via social media—a combination of posts on the study’s Facebook and Instagram pages and AI/AN community partner pages. Community partners can also directly provide contact information for potentially interested participants to RAND via our secure file sharing platform. Individuals can go online using a QR code or link to complete the screening questionnaire. There is also a toll-free 800 number potential participants can call to speak with a staff person. Basic contact info is collected online in addition to the screener. We then follow up by phone with any eligible participants and potential participants who have not completed the screener. Eligible participants are consented on the phone and complete a web-based baseline self-report survey, which takes approximately 45 min. We regard minimizing sample attrition as an essential aspect of conducting the study. Participants will complete a baseline, 3-, 6-, and 12-month survey. For each survey administration, detailed information will be obtained on how to reach the respondent (primary address, email, home phone, cell phone, parents’ phones, etc.). Figure 2 depicts participant flow through the study, and Fig. 3 contains a SPIRIT (Standard Protocol Items: Recommendations for Interventional Trials) flow diagram of the RCT schedule of enrollment, interventions, and assessments.

### Description of the intervention

#### Overview

**Fig. 4:**
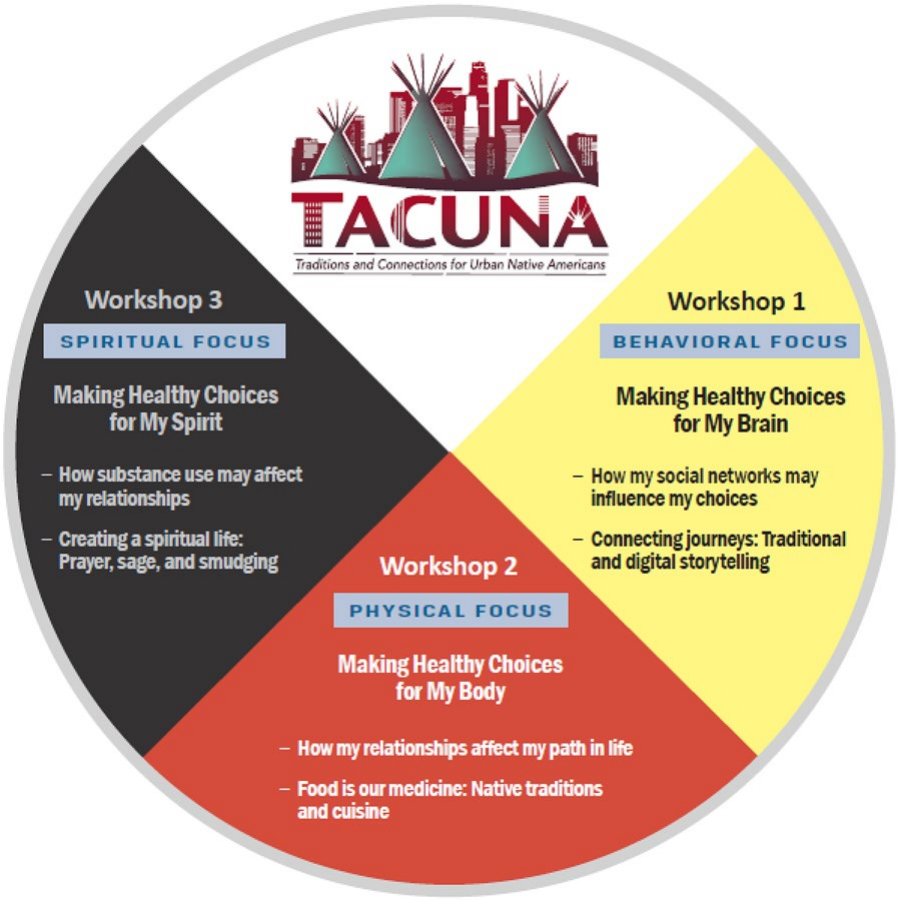
TACUNA medicine wheel

TACUNA consists of an intervention at both the individual and community level. At the individual level, we provide three workshops adapted from our previous work with urban AI/AN adolescents in our Motivational Interviewing and Culture for Urban Native American Youth (MICUNAY) study to address alcohol and cannabis use [34, 42], and our Motivational Interviewing Social Network (MISN) intervention, which augments Motivational Interviewing (MI) with personal network visualization [20]. As indicated above, workshops were adapted through focus groups with AI/AN emerging adults, parents of AI/AN emerging adults, and providers who work with AI/AN emerging adults [33]. The goal of the TACUNA workshops is to integrate MI with traditional practices and address social network factors that may affect personal choices across risk and protective behaviors (Fig. 4).

The three TACUNA workshops are all 2 h long. Table 1 provides an overview of workshop content, and Table 2 provides information on handouts provided in each workshop. The first hour of each workshop focuses on using MI to address substance use choices and social network choices; the second hour is focused on AI/AN culture and traditional practices. Workshops are interactive and use engaging handouts and videos that are discussed as a group. In addition, workshops are virtual because of COVID-19; thus, participants are sent all workshop materials via Fed Ex before they begin, including handouts, cooking ingredients for the demonstration on healthy Native cuisine, and sage for the workshop on spirituality.

**Table 1:** TACUNA workshop intervention

Workshop	Summary of activities
1	**Making healthy choices for my brain** Introduction, opening prayer, and purpose of group Generating rules for a successful group Discuss emerging adults’ substance use Social networks and choices “Your Use” and choices Medicine Wheel Historical trauma and cultural identity Telling your own story (oral and digital)
2	**Making healthy choices for my body** Introduction, opening prayer, and purpose of group Generating rules for a successful group Pros and cons of AOD use Social networks and choices The path of choices Willingness and confidence rulers around personal AOD use Medicine Wheel Discussion of Native American cooking with videos Cooking demonstration with virtual cooking for participants
3	**Making healthy choices for my spirit** Introduction, opening prayer, and purpose of group Generating rules for a successful group What can happen when people use AOD Social networks and choices Thinking ahead and making healthy choices Wheel of the Future Willingness and confidence rulers around social network choices Medicine Wheel Discussion of spiritual life Smudging ceremony

**Table 2:** Description of TACUNA Workshop Intervention Handouts

Workshop	Input	Output
1, 2, 3	Social networks diagram	Visual display of network connections
1	Facts about street fentanyl	Information about fentanyl and associated risks
1	The brain	Information about how alcohol and drugs influence brain activity
1	Opioids stimulate pleasure center	Information about how opioids affect brain functioning
1	Opioids in the body	Information about how opioids affect brain and body functioning
1	College GPA and AOD use	Information on associations between college grade point averages and AOD use
1	Drug and alcohol brochure	Information about short- and long-term effects of alcohol and drugs
1, 2, 3	Medicine Wheel	Information on the four domains
1	The dancing brain	Information about serotonin and endorphins
2	Path of choices	Information about the most current AOD prevalence of use data
2, 3	Rulers	Willingness and confidence from 1 to 10 to make a change
2	Traditional food principles	Cultural traditions around Native foods
2	Three Sisters garden planting guide	Information about planting a Three Sisters garden and its cultural significance
2	Food is our medicine brochure	Healthy Native recipes participants can try at home
2	Traditional foods for immune system support	Information about how Native foods can support immune system health
3	HIV quiz	Information about HIV and sexual health

#### Focus on social networks

**Fig. 5:**
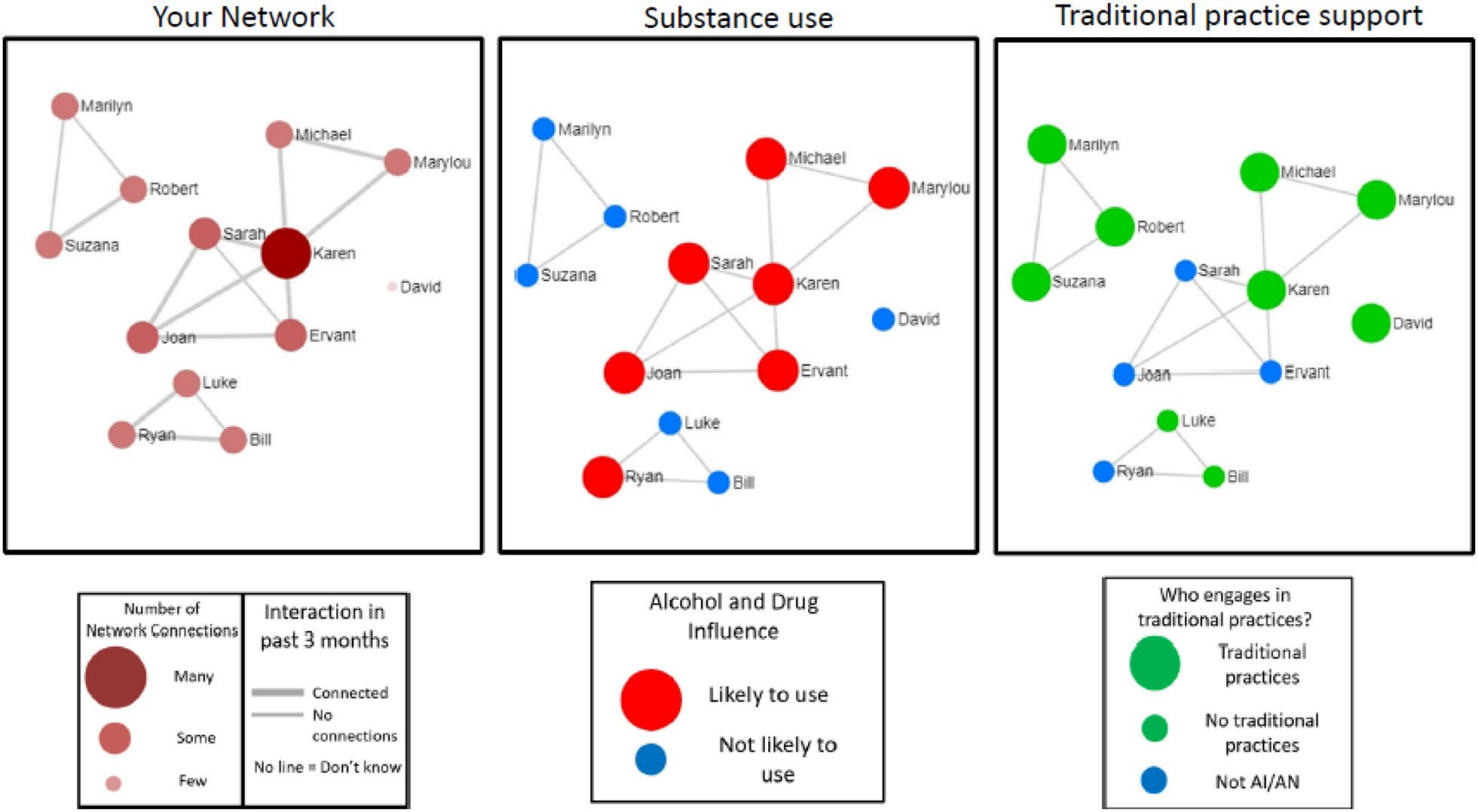
Network visualizations auto-generated using EgoWeb. Network members are represented by circles (nodes), and lines between nodes represent network contacts who interacted with each other in the past two weeks. “Your Network” visualization shows the names of people the participant reported interacting with in the past two weeks and highlights the centrality of nodes by calibrating node size and color with degree centrality (number of connections for a particular node), and line thickness with the participant’s rating of relationship strength between the two nodes. “Substance use” shows larger red nodes for people who the respondent rates as likely to use AOD in the next two weeks and smaller blue nodes for those who are unlikely. Finally, “Traditional Practice Support” shows larger green nodes for people who engage in traditional practices, and smaller blue nodes for people who do not

Using the software EgoWeb 2.0 (egoweb.info) developed by RAND researchers [20, 43, 44], participants are shown images of their network composition (who is in the network) and structure (who is connected to whom) to make them aware of positive and negative aspects of their virtual social worlds and ways other people can protect or influence them. These images are new and interesting, but also easily understood by non-experts [45]. Figure 5 depicts network visualizations that an emerging adult would see during the TACUNA workshops. Visualizations are auto-generated using EgoWeb from a participant’s network survey (see “Measures”). Each diagram will be personalized for each participant to view during the workshop.

#### Facilitation of workshops

TACUNA workshops are designed to be facilitated by Native American individuals who have strong cultural knowledge, have received training on both the curriculum and the use of motivational interviewing, and have demonstrated proficiency in delivering the workshop material. As with our other MI intervention RCTs, facilitators do not need to have a specific college degree or education background to fill this role [34, 46, 47]. We will monitor fidelity to the protocol and MI throughout the study as all workshops will be digitally recorded, and supervision will be provided weekly.

#### Workshop content

The first hour of Workshop 1 focuses on social networks and choices (Fig. 4), how substance use affects the brain, and how to make healthy choices around substance use. The second hour of Workshop 1 discusses historical trauma, cultural identity, and storytelling (specifically, how to tell one’s own personal story), and focuses on the emotional and mental dimensions of the Medicine Wheel. The Medicine Wheel is a sacred symbol that has been used by various tribal groups for many generations. It addresses the physical, spiritual, emotional, and mental aspects of wellness, and holds many of the teachings and wisdom of Native American culture [48–51]. Thus, the Medicine Wheel helps to serve as a guide for TACUNA participants to learn more about their culture, thereby helping to enhance their cultural identity.

**Fig. 6:**
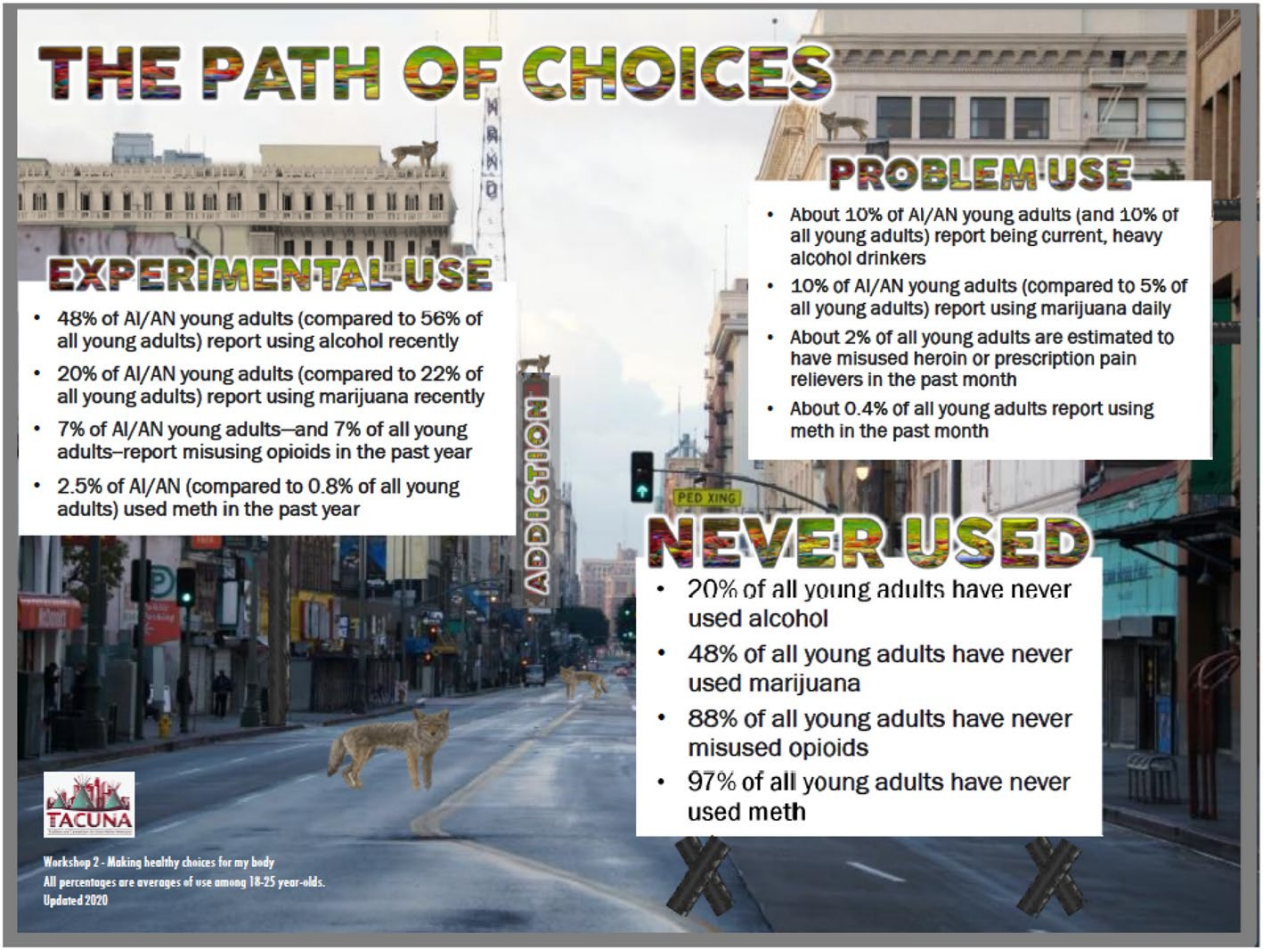
Path of Choices handout

**Fig. 7:**
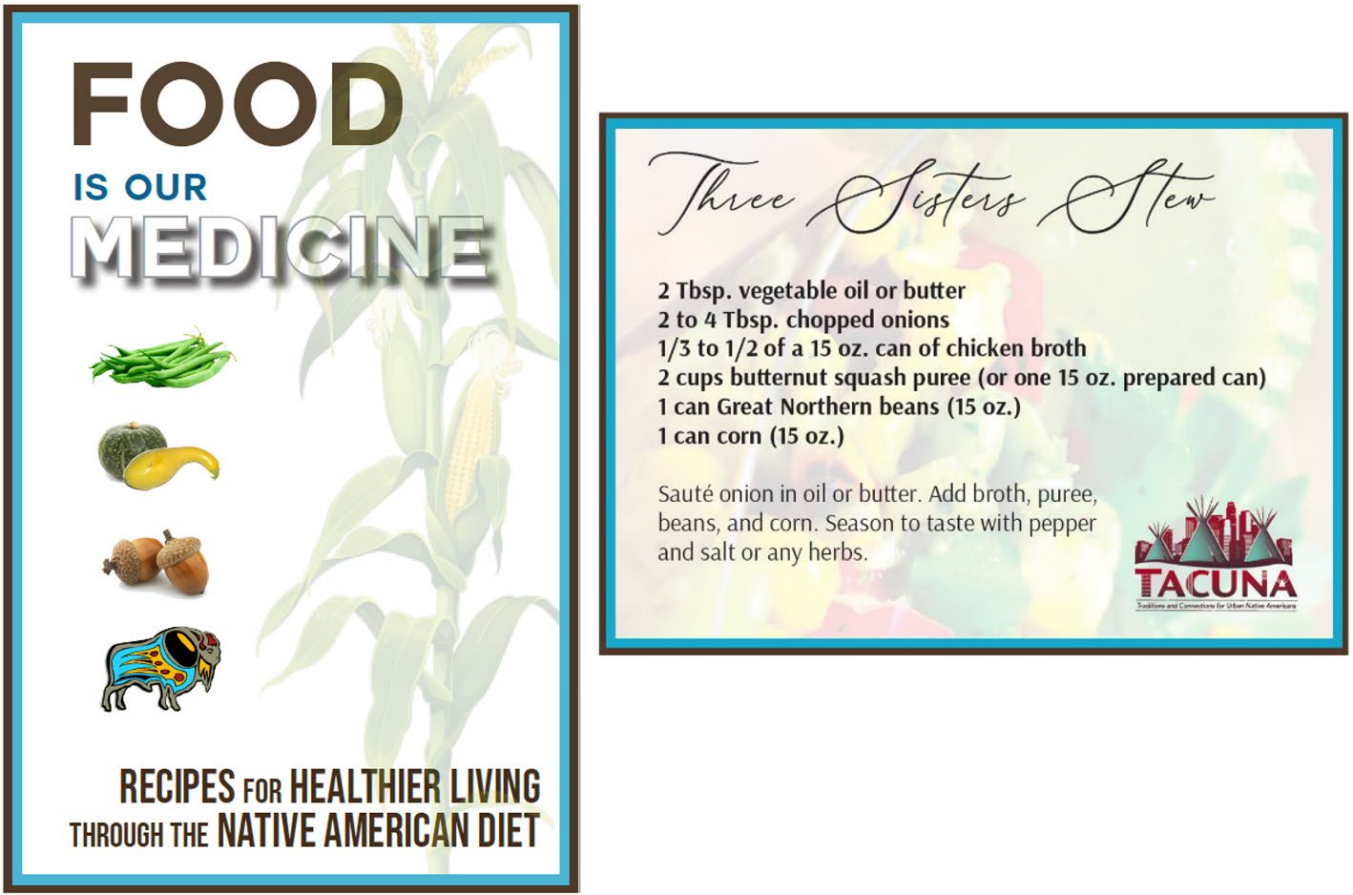
Food brochure cover and Three Sisters Stew recipe card

**Fig. 8:**
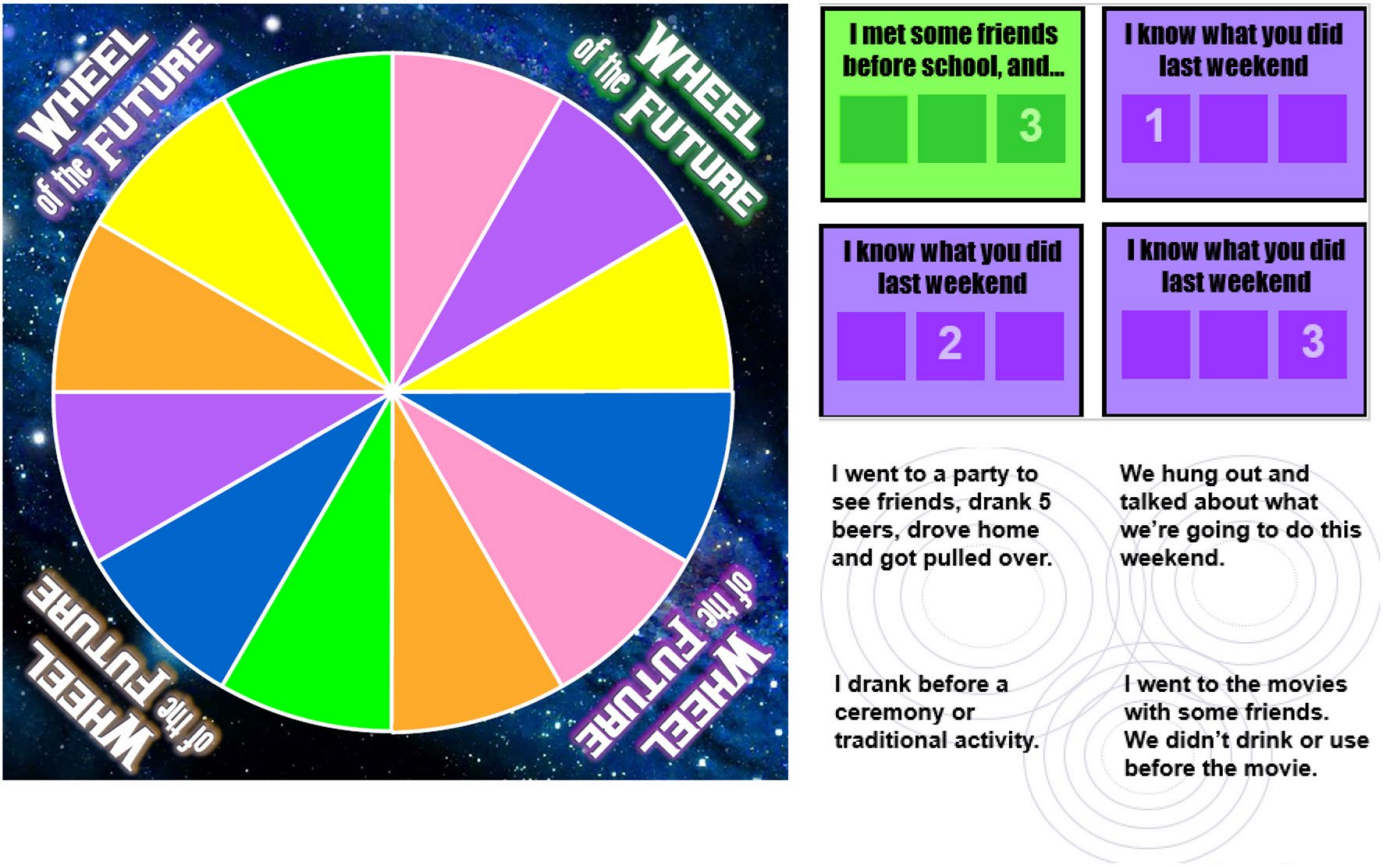
Wheel of the Future

The first hour of Workshop 2 focuses on the pros and cons of substance use, how social networks affect choices around use, the “path of choices” where people may go from experimental use to problem use to addiction (Fig. 6), and willingness and confidence to make healthy choices around substance use. The second hour of the workshop focuses on the physical dimension of the Medicine Wheel and making healthy choices about diet. The facilitator engages with the group in a cooking activity of a traditional Native American recipe, Three Sisters Stew (Fig. 7), and all participants cook in their own kitchens with the facilitator instructing virtually. As noted earlier, participants receive ingredients (paid for by the project) before the workshop so they can cook with the facilitator during the demonstration. The third workshop focuses on discussing what happens when people use substances, how social networks may affect these choices, and how to plan ahead and make healthy choices regarding substance use. The facilitator leads a game, the “Wheel of the Future,” [52] where participants virtually spin the wheel with different scenarios (e.g., got drunk at a party and drove home) and discuss how these scenarios might affect their future goals (Fig. 8). The second half of workshop 3 focuses on the spiritual dimension of the Medicine Wheel, and then focuses on prayer and the four sacred plants (cedar, sweetgrass, sage, and tobacco); the facilitator ends by leading a smudging ceremony. Smudging is a ceremony that involves burning sacred plants to help cleanse oneself, clear away negative energy, and connect ourselves with our spirit and Creator. As noted, participants receive sage before this workshop, which is gathered, bundled, and shipped by our community partner, SPIWC.

#### Wellness circles

**Fig. 9:**
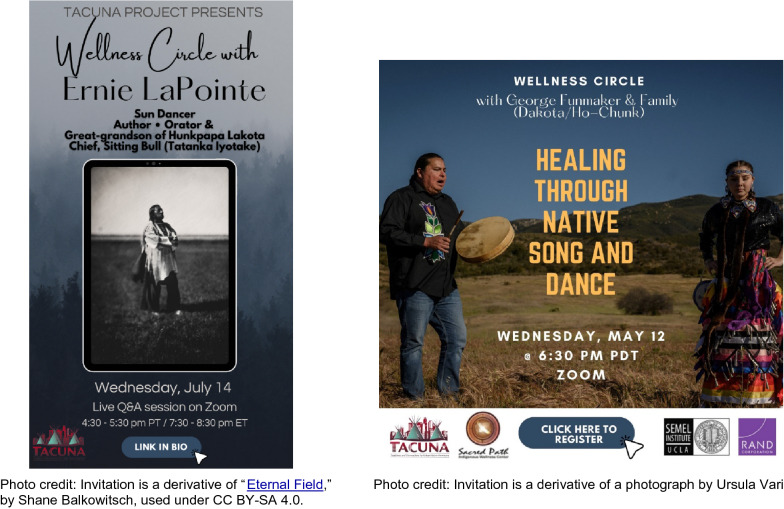
Wellness circle invitations

At the community level for the TACUNA intervention, we offer wellness circles (WC) approximately once a month that participants will attend and invite members of their social networks to (Fig. 9). The WC is focused on bringing people together to celebrate health, wellness, and tradition. The WC will also focus on how social networks and cultural connectedness can influence healthy behaviors. Some examples of WC activities include a Native American chef teaching how to cook different Native recipes, a focus on Native American dances and their meaning, education about how Native people came to be in California, and discussion of the Medicine Wheel and how to incorporate these teachings into one’s life.

#### Opioid education workshop

**Fig. 10:**
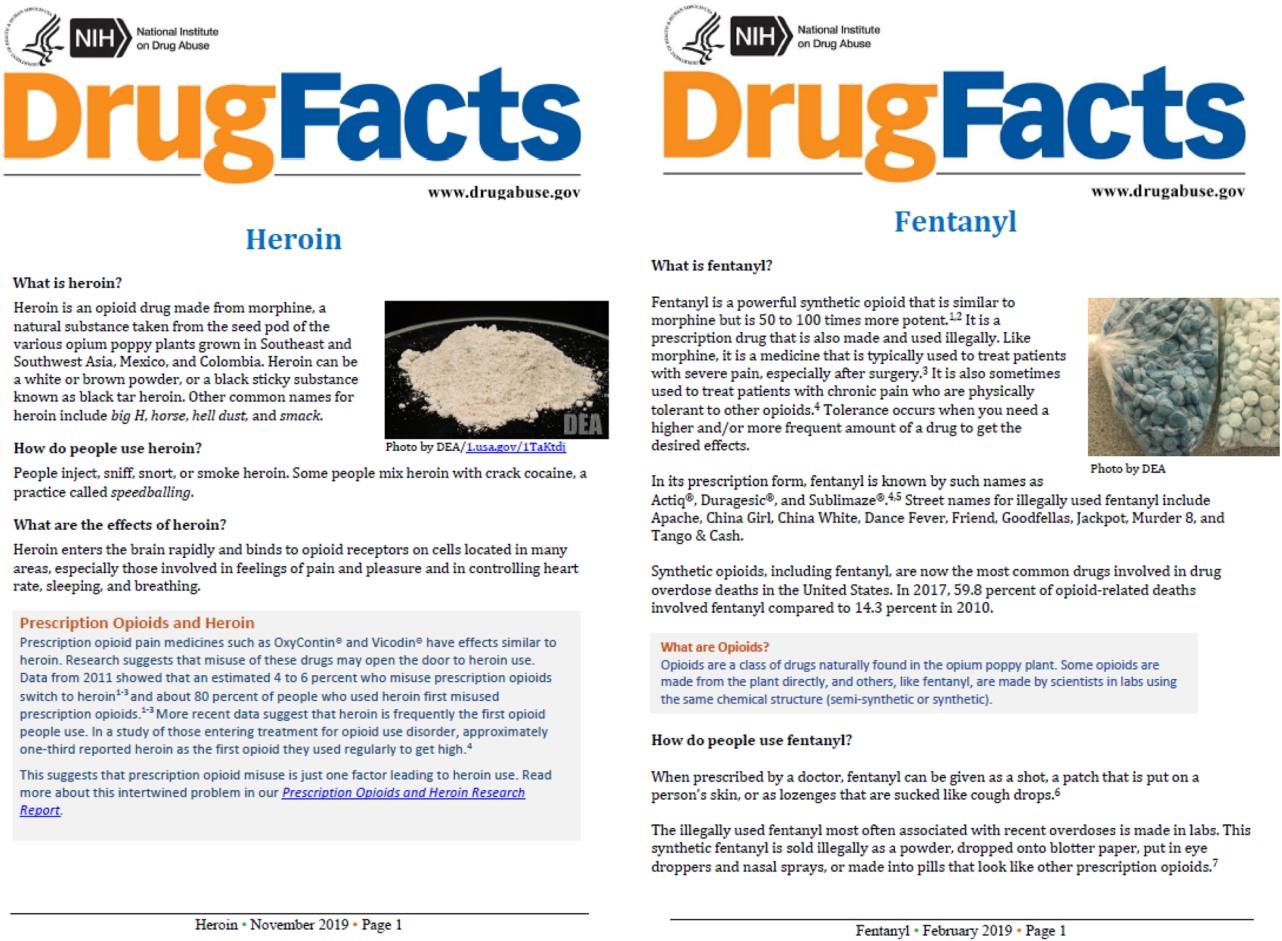
Opioid education workshop, general overview of opioid handouts

**Fig. 11:**
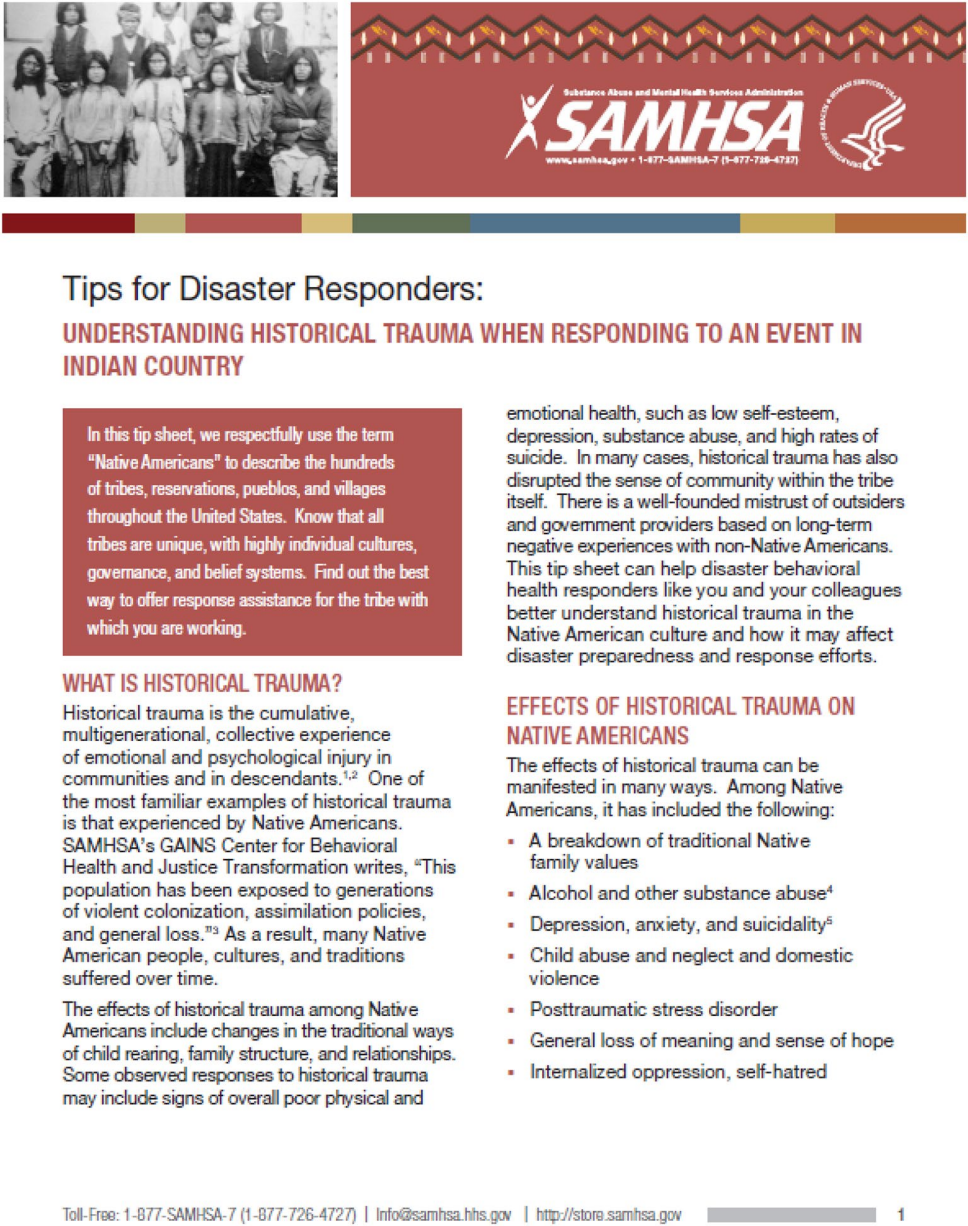
How the opioid epidemic has affected AI/AN communities handout

We will compare TACUNA to a culturally appropriate opioid education workshop taken from prevention and education materials recommended by the National American Indian & Alaska Native Addiction Technology and Transfer Center, which is funded by the Substance Abuse and Mental Health Services Administration (SAMHSA) [53]. This two-hour workshop focuses on providing a general overview of opioids (Fig. 10), how the opioid epidemic has affected AI/AN communities (Fig. 11), treatment for opioid use disorders, physical health and pain self-management, and how involvement in cultural traditions can be protective. Table 3 provides a summary of content, and Table 4 provides information on handouts provided in each workshop. This workshop is designed to be facilitated by Native American individuals with strong cultural knowledge who have received training on the curriculum and have demonstrated proficiency in delivering the workshop material.

**Table 3:** Opioid control condition

Part	Summary of activities
**1**	**Opioids: General overview** Introduction, opening prayer, and purpose of group Generating rules for a successful group Icebreaker Heroin overview Fentanyl overview Video Discussion
**2**	**American Indians/Alaska Natives and the opioid epidemic** Responding to the opioid Crisis: An update for Tribal leaders Historical trauma Video Discussion
**3**	**Treatment for opioid use disorders** Medications for opioid use disorders At home detoxification for opioids only Video Discussion
**4**	**Culture, physical health, and pain self-management strategies** Physical wellness checklist Pain self-management techniques Video Discussion

**Table 4:** Description of opioid control condition handouts

Part	Input	Output
1	NIH drug facts—Heroin	Information about heroin and associated risks
1	NIH drug facts—fentanyl	Information about fentanyl and associated risks
2	Responding to the opioid Crisis: An update for Tribleleaders	Information about the opioid epidemic in AI/AN communities
2	Understanding historical trauma when responding to an event in Indian country	Information about historical trauma and substance use in AI/AN communities
3	Workplace solutions: Medication-assisted treatment for opioid use disorder	Information about opioid use disorder and medications for OUD treatment
3	At home detoxification from opioids only	Information about recognizing withdrawal symptoms and supporting a friend or loved one who may be detoxing from opioids
4	Your healthiest self: Physical wellness checklist	Information about positive physical habits that individuals can follow to achieve and maintain a healthy lifestyle
4	Pain self-management strategies	Information about pain self-management strategies that do not involve narcotic pain relievers

### Analysis plan and data management

Data at all time points (baseline, 3-, 6-, and 12-month) will be collected using web-based surveys. We will conduct descriptive statistics and examine missing data. Frequencies will be examined for evidence of sparseness for categorical data and for non-normality (using plots, examination of skewness, kurtosis, etc.) for continuous variables. Where sparseness exists in categorical variables, we will collapse as necessary to produce cell sizes sufficient for analysis. Where non-normality is evident, variables may be transformed. Outliers may be recoded or omitted if necessary. Missing data will be dealt with using multiple imputation and/or full information maximum likelihood estimation. The N of 370 was determined in a priori power analyses to be sufficient to detect small to moderate intervention effect sizes for all primary and secondary outcomes. We chose to use the final projected sample size accounting for 20% attrition at the 12-month at follow up (n = 148 per arm). Within a repeated measures framework, these sample sizes, assuming a correlation between repeated assessments of 0.50, four timepoints, and alpha of 0.05, we have 80% power to detect a standardized effect size (d) of 0.31 between groups and 0.22 within groups. For mediation models, using bias-corrected bootstrapping, there is 80% power to detect a mediated/indirect effect when δ = 0.28 (i.e., effect of the predictor on the mediator) and β = 0.26 (i.e., effect of the mediator on the outcome) (Additional file 1).

All data collection and Record Management System functions will be conducted on the RAND Survey Research Group’s secure network segment. Computers on the secure network segment are isolated from the rest of the RAND network (e.g., no Internet access, e-mail, or file sharing between these computers and the unclassified network), minimizing the possibility of infection by malicious software and unintentional exposure of sensitive data. The computers on the segment will also employ standard password protection along with file and folder permissions limiting access to appropriate project staff.

#### Baseline equivalence across experimental groups

We will evaluate comparability of experimental groups with respect to potential confounders. Categorical methods of analysis (e.g., cross tabulations, chi-square) will be used to compare groups for discrete data (e.g., employment, school status). ANOVA or t-tests will be used to test for homogeneity of groups for continuous data at baseline. If a statistically significant difference is found, the covariates will be included in all subsequent analyses. If we observe considerable differences in the intervention and control groups that cannot be adequately accounted for with the addition of model covariates, we will develop analytic weights using propensity methods to balance the groups.

### Aims of the project

We plan to compare our active control group (i.e., those who receive the opioid education workshop) to participants who receive TACUNA and the WC. We will compare outcomes between the two groups at 3, 6, and 12 months to determine (a) whether changes occur in initiation and escalation of opioid use and alcohol and other drug use, such as cannabis, and related consequences; time spent around peers who use opioids, cannabis, and alcohol; and perceived prevalence of peer use, (b) whether clinically significant changes occur in physical, social, emotional, and functional well-being, as well as spirituality and cultural connectedness, and (c) if reductions occur, estimate effect sizes. In addition, we will explore potential mechanisms of change for decreases in use through mediation analyses, including changes in social networks and cultural connectedness.

### Screener

Participants will be screened with the Rapid Opioid Dependence Screen, an 8-item measure of opioid dependence based on the Diagnostic and Statistical Manual of Mental Disorders, Fourth Edition criteria, and designed for quick, targeted screening in clinical and research settings (e.g., use more opioids to get the same high as when first started using opioids, worry about use, find it difficult to stop use) [54].

### Primary outcomes

#### Frequency of opioid, alcohol, and cannabis use

We will assess *substance use history* at each assessment with Monitoring the Future (MTF) items [55]. The consistency and reliability of these measures have been shown in numerous studies [56–58]. At baseline, we will measure *lifetime* (0 = 0 times, 1 = 1 or 2 times, 2 = 3–9 times, 3 = 10–19 times, 4 = 20–39 times, 5 = 40–99 times, 6 = 100 + times), *past year*, *3-month* (0 = none, 1 = 1 time, 2 = 2 times, 3 = 3–5 times, 4 = 6–9 times, 5 = 10–19 times, 6 = 20–30 times, 7 = 31 + times) and *30-day use* (number of days). At follow-up time points, we will measure past 3-month and past 30-day use.

### Secondary outcomes

#### Cultural connectedness

*Cultural connectedness* will be measured with the Cultural Connectedness Scale, which comprises 29 items that address three dimensions: identity, traditions, and spirituality [59]. Respondents answer 11 yes/no questions (e.g., “I have a traditional person, elder, or other person who I talk to”), and use a scale from 1 = “strongly disagree” to 5 = “strongly agree” for 18 items (e.g., “I feel a strong connection/attachment towards my Native American community or Tribe”).

#### Social networks

*Social network composition and structure*. Participants will complete network interviews at baseline and follow-up to measure network characteristics and changes using procedures from our previous work [60, 61] and standard procedures for collecting and analyzing personal networks [20, 62, 63]. Participants will be asked to name up to 15 network contacts (“alters”) who are at least 18 years of age or older to produce unbiased network measures [64, 65]. Participants will answer questions about each alter (e.g., demographics, relationship quality, likelihood to use drugs) to produce raw data for network composition measures (e.g., % who engage in heavy drinking) [66]. Participants will identify ties among alters to produce raw relationship data to measure network structure (density of ties, average centrality of network members who use substances, etc.) [67, 68].

### Economic outcomes

The economic evaluation is planned as a within-trial cost-effectiveness analysis to assess the incremental cost and effectiveness of the TACUNA intervention compared with the opioid education control condition. Following recommendations from the Panel of Cost-Effectiveness in Health and Medicine [69], we plan to report costs and cost-effectiveness estimates in terms of two reference cases, one based on a program perspective (programmatic and provider time costs) and the other on a limited societal perspective that incorporates a broader range of costs, such as opportunity costs to participants.

#### Resource use and costs

We will account for programmatic costs following the micro-costing approach of prior work [70–73]. Resource use and unit prices will be obtained from several sources (e.g., program records, primary collection of time-use data, and published salary values from government websites) and collected alongside the trial using a standardized tool created through collaboration with the HEAL Prevention Initiative Cooperative Economic Workgroup. While the analysis will exclude research and development costs, all other programmatic costs will be classified separately by the trial condition to which they apply (i.e., TACUNA or opioid education workshop) as well as the phase of implementation of the intervention (i.e., start-up or intervention delivery). Start-up costs will include those costs accrued prior to intervention delivery, including labor and materials costs associated with hiring and training facilitators, pilot-testing of the virtual delivery of the intervention, and planning meetings with the Elder Advisory Board to refine and tailor the interventions. Intervention delivery costs include time and labor for scheduling workshops and wellness circles, preparation and delivery of the interventions, and ongoing management, as well as direct costs of materials (e.g., food, sage, handouts) and shipping. For the limited societal perspective, we will also incorporate opportunity costs to participants based on participant-level attendance records and earnings information collected through the participant surveys [69, 70]. We had initially planned to track space and utility costs, as well as transportation costs; however, the shift to virtual implementation of the interventions precludes the accrual of costs in these domains.

### Effectiveness

We will follow recommendations for economic evaluations of AOD use interventions [74, 75] and consider multiple outcomes to compare effectiveness at 6 and 12 months after baseline. Our primary effectiveness measures will be abstention from opioid use in the past 3 months (either through preventing initiation or discontinuing use); abstention or consumption below the threshold of heavy use for alcohol and cannabis [46]; and a reduction in time spent around peers who use opioids. Because our focus is on prevention, an effectiveness outcome related to peers will be informative.

### Analysis

#### Primary and secondary outcomes

Our analytic approach will focus on our primary and secondary outcomes. We will compare opioid education workshop participants to TACUNA participants, with covariates and the baseline measure of the outcome of interest. Given the longitudinal design, we may use more than one method for analyzing the data. SAS Proc Glimmix may be used with overdispersion and/or zero-inflation accounted for as needed in the modeling approach using restricted maximum likelihood estimation. Alternatively, we will work within a multigroup latent growth model framework using maximum likelihood estimation for continuous outcomes or weighted least square mean and variance (WLSMV) for categorical outcomes as implemented in Mplus. Outcomes will be tested at each time point [3, 6, and 12 months), and longitudinal analyses will be used to test for differences in change over the 12-month period. Testing for differences at each time point provides estimates of the intervention effect at that time; however, the longitudinal analysis will capture patterns of change over time; that is, whether the average change on outcomes within each group will produce different trajectories. To enhance interpretability of our results, we will use estimated differences between groups to calculate standardized effect sizes in terms of *d* (for continuous variables); we will use odds ratio estimates to calculate risk difference and number needed to treat, based on outcome proportions for the opioid education workshop group. All primary analyses will be by intention to treat, and we will attempt to follow up with all individuals, regardless of attendance. We will also model attendance to detect factors that alter the probability of attending using ordinal logistic regression approaches. Although not part of the design, it is possible that individuals in the control group may attend a wellness circle. We will be able to include this as a covariate in our models.

#### Mechanisms of change

We will conduct mediation analyses to examine whether changes in social networks and cultural connectedness serve as explanatory mechanisms for reductions in opioid, cannabis, and alcohol use. This will allow us to leverage our novel dataset to explore predictors of behavior change, thereby improving the development of future interventions. We will first conduct a multivariate mediation test to determine if the total mediation effect is statistically significant to control Type I error rate. If the total mediated effect is significant, we will then examine individual mediators to determine statistical significance. The estimate of the effect of the intervention on the outcome can be divided into the direct effect and the indirect (mediated) effect. The standard errors of the individual indirect effects will be estimated using bias-corrected bootstrapping.

#### Economic outcomes

*Economic analysis.* We will conduct an intent-to-treat analysis. We will first perform deterministic analyses (i.e., direct calculations) to sum intervention costs (and opportunity costs for the limited societal perspective), yielding incremental net costs as the difference between net costs per participant to (a) those receiving TACUNA and (b) those receiving the opioid education workshop. Next, we will perform probabilistic (Monte Carlo) analyses to account for uncertainty and variability in cost parameters. We will construct short-term incremental cost-effectiveness ratios (ICERs) for each outcome, which reflect the marginal cost associated with achieving an additional unit of the effectiveness outcome through TACUNA, relative to the opioid education workshop control condition. We will assess model and parameter uncertainty on each outcome’s effect sizes, allowing us to consider a range of values from findings related to uncertainty or variability in the relative effectiveness of TACUNA compared to the opioid education workshop. We will construct ICER confidence intervals using non-parametric bootstrapping techniques within the multivariable framework, although we may consider other methods [76–81].

##### Sensitivity analysis

We will perform sensitivity analyses to test the importance of key model parameters, such as modifying the price of a given resource or excluding start-up costs from calculations, on ICER findings. Additionally, we will consider how costs and cost-effectiveness differ under alternative parameter assumptions as well as under different assumptions about attendance or treatment dosage.

#### Limitations and alternative methods considered

There are some important limitations to our work. First, we had to quickly change our implementation method from in-person groups to virtual groups because of the COVID-19 pandemic. While virtual implementation is potentially a strength as it lowers some barriers to entry, such as travel, a known barrier to research participation among underserved populations in urban areas [82, 83], it is quite challenging to recruit and manage retention during a pandemic. A second limitation is that we are adapting the MISN from use with individuals to a group context. However, our initial use of the MISN in the three pilot workshops in phase 1 highlighted that the emerging adults found discussion of social networks in the group engaging and enjoyable; thus, we expect the adaptation to be successful in the RCT. Third, we developed the manual with California samples as we originally planned to conduct the study only in California. Now that the interventions are virtual, we are recruiting across the U.S., and other states’ urban areas may differ from California’s urban areas. It is important to note, however, that even in our urban areas in California, there was wide diversity among focus group participants, with over 50 tribes represented, and with emerging adults in varied urban areas highlighting different issues (e.g., greater emphasis on housing and job opportunities in Northern California versus parenting and urban sprawl in Southern California; greater emphasis on migrating among reservations or rancherias, rural areas, and urban areas in Northern California). Given this diversity, it is likely that the program will have broad appeal, and we are creating a template for how to make the program accessible to a wider population because of the more flexible online format. Fourth, we are not using a neutral control group, thus conclusions about efficacy of the intervention are in reference to our active control group. However, our focus group work in these communities and discussions with our Elder Advisory Board and SPIWC emphasized the importance of ensuring that all participants in the RCT received some type of cultural programming. Finally, our economic analysis does not adopt a full societal perspective, as collecting the full range of potential cost savings from reduced healthcare visits, or criminal justice involvement was not feasible in our study context (i.e., we are unable to link participants to administrative healthcare or law enforcement data sources). Reduced substance use and initiation may generate cost savings in sectors that we are unable to capture; thus, our cost-effectiveness analysis may be conservative. However, our programs focus on both prevention and intervention, and our target population is likely lower-risk in terms of these outcomes. Thus, this is unlikely to substantively influence our within-trial economic evaluation, although it may have implications for longer-term budget impact analyses.

## Discussion

This is the first study to date to address opioid use among urban AI/AN emerging adults by testing a brief group MI intervention that integrates traditional practices and social network visualization. The TACUNA intervention involves workshops at the individual level, a wellness circle at the community level, and is designed to reduce opioid, alcohol, and cannabis use, increase cultural connectedness, and encourage healthy social networks among urban AI/AN emerging adults.

This clinical trial will compare the effectiveness of the TACUNA intervention to a culturally sensitive opioid education workshop, and examine outcomes over a 12-month period. We will assess not only the effects of these two culturally appropriate interventions on subsequent substance use, but we will also examine whether cultural connectedness and support from social networks mediate any effects of the intervention. This is important as urban AI/AN emerging adults face numerous challenges given experiences of acculturative stress and the disconnection from their cultural community [12, 18, 84, 85]. Previous work has emphasized the protective effects of culture in addressing health disparities among this population [28, 86, 87], and in promoting resilience for AI/AN people living in urban areas [88]. Further, there is only one study to date assessing AI/AN youth social networks, and participants were younger and lived in remote villages across the state of Alaska [89]. Findings indicated that connections to adults and Elders were protective, highlighting how social networks and community engagement foster resilience [89]. The current study will be able to address important questions about the protective role of social networks for urban AI/AN emerging adults that have not been previously examined, providing an understanding of the amount of support these emerging adults receive to engage in traditional practices and make healthy choices around substance use. In addition, by incorporating an economic evaluation within the broader study, we can also inform key questions related to feasibility, affordability, and economic efficiency. In sum, the current study addresses a critical programming need for urban AI/AN emerging adults as it focuses on increasing resilience through reduction of substance use, provision of cultural resources, and increased supportive social connections.

## Supplementary Information

**Additional file 1.** SPIRIT 2013 checklist.

## Data Availability

Once collected, de-identified data from this study will be available from the corresponding author on reasonable request one year after all aims of the project are completed. Requestors of data will be asked to complete a data-sharing agreement that provides for (1) a commitment to using the data only for research purposes and not to identify any individual participant or tribal affiliations; (2) a commitment to securing the data using appropriate computer technology; and (3) a commitment to destroying or returning the data after analyses are completed. Data requests for this study must be made to the TACUNA Urban Intertribal Native American Reivew Board. Review of data requests by the TACUNA Urban Intertribal Native American Reivew Board is crucial to ensure that AI/AN communities are protected from potential harm or misuse of data.

## Abbreviations

AOD: Alcohol and other drugs
AI/AN: American Indian/Alaska Native
HEAL: Helping to End Addiction Long-term
ICER: Incremental cost-effectiveness ratio
IRINAH: Intervention Research to Improve Native American Health
MTF: Monitoring the Future
MI: Motivational interviewing
MICUNAY: Motivational Interviewing and Culture for Urban Native American Youth
MISN: Motivational interviewing social network
MOUD: Medications for opioid use disorder
OUD: Opioid use disorders
RCT: Randomized controlled trial
RFA: Request for applications
RODS: Rapid Opioid Dependence Screen
SAMHSA: Substance Abuse and Mental Health Services Administration
SPIWC: Sacred Path Indigenous Wellness Center
SPIRIT: Standard protocol items: recommendations for interventional trials
TACUNA: Traditions and Connections for Urban Native Americans
WC: Wellness circle
WLSMV: Weighted least square mean and variance
